# Supplementary material for: Planktonic Aggregation Enhances Antibiotic Tolerance in Non-MDR Acinetobacter baumannii
Source: Microorganisms. 2025 Dec 19;14(1):8. doi: 10.3390/microorganisms14010008 (PMC12843791; doi:10.3390/microorganisms14010008)
Supplement: Supplementary file 1 [file microorganisms-14-00008-s001.zip › microorganisms-3992882-supplementary.pdf]

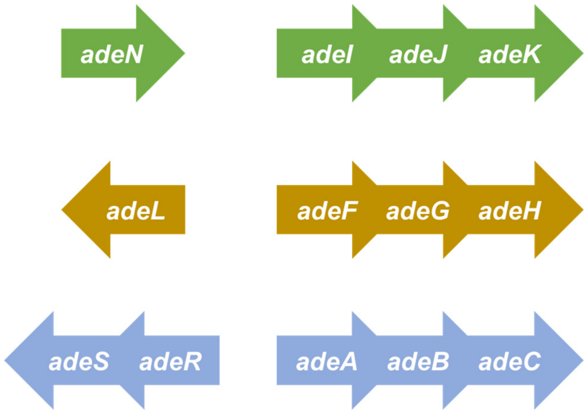

Figure S1. Schematic diagram of the genetic organization of core RND efflux pump operons in *Acinetobacter baumannii.*

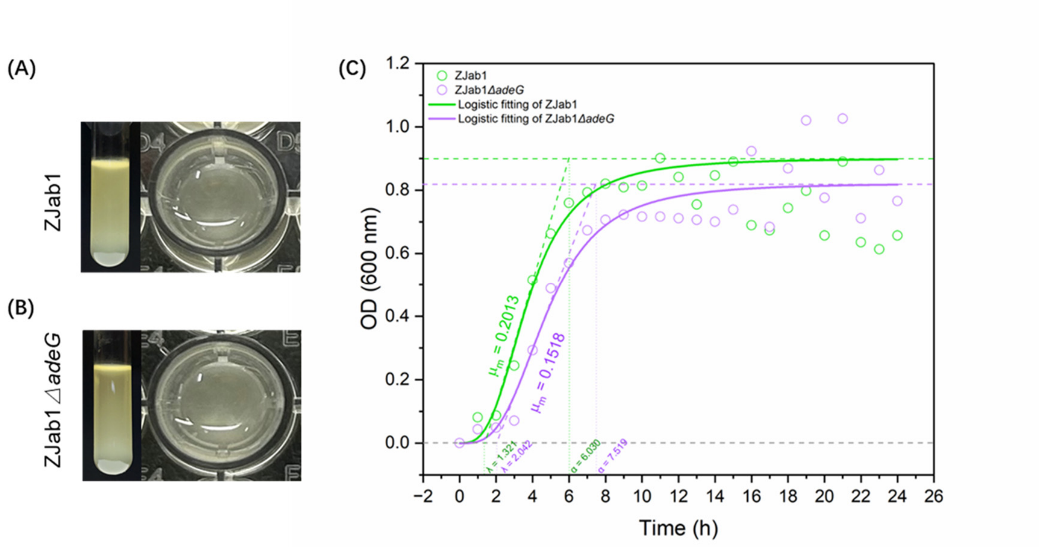

Figure S2 Growth and Aggregation Phenotypes of ZJab1 and ZJab1*ΔadeG*. (A) and (B) show the side view of ZJab1 and ZJab1*ΔadeG* cultures in glass tubes and the top down view of 96-well microplate culture, respectively. (C) Growth curve and lag phase difference analysis under dynamic culture conditions. λ denotes the end time point of the lag phase, and α denotes the end time point of the logarithmic growth phase. μ_m represents the maximum growth rate.

Table S1 Bacterial strains and plasmids used.

Strain and plasmid	Genotype or Description	Source
HFab1~HFab104	Clinical isolates	Lab stock
YZUMab17	Clinical isolates	Lab stock
YZUMab17*ΔadeG*	*adeG* gene knockout in YZUMab17	This study
ZJab1	Clinical isolates	Lab stock
ZJab1*ΔadeG*	*adeG* gene knockout in ZJab1	This study
pKD4	Provide resistance screening marker (KanR)	Lab stock
pAT04	Promotion of homologous recombination	Lab stock
pAT03	Delete the kanamycin resistance gene sequence	Lab stock

Table S2 Primers for detection of the RND efflux pump

Target gene	Primer sequence (5'–3')	Length (bp)	Functional description	Source
adeA	F: GGCGTATTGGGCAATCTTTTGT<br>R: GTCACCGACTTTCAAGCCTTTG	524	Membrane fusion protein, involved in antibiotic efflux	(1)
adeB	F: CGGAAGGCATGGAGTTTAG<br>R: TACTGCCGCCAATACCAG	404	Transporters, which bind directly to and excrete antibiotics	
adeC	F: GACAATCGTATCTCGTGGACTC<br>R: AGCAATTTTCTGGTCAGTTTCC	1331	Outer membrane channel proteins form transmembrane channels to complete antibiotic efflux	
adeS	F: GTGGACGTTAGGTCAAGTTCTG<br>R: TGTTATCTTTTGCGGCTGTATT	531	Histidine kinase sensor	
adeR	F: TCACATGGCTATCTACGGTTGG<br>R: TGAAGGCATGAGTGTTATTCGG	533	Response regulator	
adeF	F: GGTGTCGACCAAGATAAACG<br>R: GTGAATTTGGCATAGGAACG	208	Transport protein	This study
adeG	F: GGTTCTGGTGACTACGCAATG<br>R: CCGCGATATAGGCATCTTG	1471	Membrane fusion proteins mediate substrate transfer from the intima to the outer membrane	
adeH	F: CGATCAGCAAATTCAGGCTC<br>R: GCTTGCAATGATTTGGTTAC	181	Outer membrane channel proteins, which assist in the excretion of antibiotics outside the cell	

adeL	F: GACGGTGATGCTTATATCGACT<br>R: CAACAAGTATCGCGCTTG	140	Transcriptional regulation factor	
adeI	F: CAACAAGTATCGCGCTTG<br>R: CTTAGTTGTTGACGTAAACGC	661	Transporters, efflux broad-spectrum substrates	(2)
adeJ	F: GCGGTCATTAATATCTTTGGC<br>R: CGTTAATGTATGAGCCACCC	1144	Membrane fusion protein enhances substrate recognition and efflux efficiency	
adeK	F: CAAGTTGCTCAAGCATGG<br>R: CTAGATGGTTGGTGTACCAC	917	Membrane fusion protein enhances substrate recognition and efflux efficiency	
adeN	F: GAGCTAGGCATGGATTCTG<br>R: CACGTGCTGAATCTTCAATC	1387	Transcriptional regulation factor	

Table S3 Primers for the construction of the mutant strain

Name	Primer Sequence (5'–3')	Length (bp)	Functional description
AdeG-UP-WF	<u>GGGCGGATATCTCGAG</u>GGTTCTGGTGACTACGCAATG	175	*adeG* upstream sequences amplification
AdeG-UP-F	GGTTCTGGTGACTACGCAATG		
AdeG-UP-R	CCTTGAGCATTGACTGAAAGC		
AdeG-DOWN-F	GCCTGGTGTAGAAAGTGCTG	188	*adeG* downstream sequences amplification
AdeG-DOWN-R	CCGCGATATAGGCATCTTG		
AdeG-DOWN-WR	<u>GCGGTACCCGGGATCC</u>CCGCGATATAGGCATCTTG		
AdeG-F	GCGGTGCTCAACGTAAAAG	1471	Recombineering screening primers
AdeG-R	CAAGTTGCAGTTTAAAGCCG		
Kan-AdeG15-F	<u>AGTCAATGCTCAAGG</u>TGTGTAGGCTGGAGCTGCTT	1478	Kanamycin resistance fragment amplification
Kan-AdeG15-R	<u>CTTTCTACACCAGGC</u>CATATGAATATCCTCCTTAG		

Primers were designed to harbor extensions homologous to ~15 bp (underlined) of the target gene. F, forward; R, reverse

Table S4. Primers for quantitative real-time PCR

Target gene	Primer Sequence (5'–3')	Length (bp)	Molecular function	Source
rpoB	F: ATGCCGCCTGAAAAAGTAAC<br>R: TCCGCACGTAAAGTAGGAAC	155	Housekeeping gene	(3)
csuA/B	F: CTCAAGCAGCTGTTACTGGTC<br>R: CAGGATCTGTTCCGTCACAAG	213	Chaperone Usher (CU) pili assembly system, a family of proteins involved in Type IV pili assembly	(4, 5)
csuC	F: TTACACCTGTGATGGCACAAGC<br>R: GCATATGCTTCTCGCCTGC	234		
csuD	F: GCCTCCACCACCAAGAGAC<br>R: CCAATCACTCGCATCACCAC	253		
csuE	F: TCAGACCGGAGAAAAACTTAACG<br>R: GCCGGAAGCCGTAT GTAGAA	150		
pilA	F: GCAGCTTGCTGCCGAATAC<br>R: GAGCATTGTCTGGTTCAGACG	203	Type IV Pilus (T4P), forming and assembling Type IV pilus	(5)
pilB	F: CTATCGGGTGCGCTATC<br>R: CATTGCGCTTGATGGGTC	253		
pilY	F: GGTATGCCGTCTGTTCAAGC	284		

	R: TCCACTTCGGCTGTCGCC	
pilW	F: GTAGCTGCTGCAACACAGC	256
	R: GGCGTTGTATCTGTTAATTGTGG	

F, forward; R, reverse

REFERENCES:

1. Jia W, Li C, Zhang H, Li G, Liu X, Wei J. 2015. Prevalence of Genes of OXA-23 Carbapenemase and AdeABC Efflux Pump Associated with Multidrug Resistance of *Acinetobacter baumannii* Isolates in the ICU of a Comprehensive Hospital of Northwestern China. Int J Environ Res Public Health 12:10079-92.
2. Li S, Duan X, Peng Y, Rui Y. 2019. Molecular characteristics of carbapenem-resistant *Acinetobacter spp.* from clinical infection samples and fecal survey samples in Southern China. BMC Infect Dis 19:900.
3. He X, Lu F, Yuan F, Jiang D, Zhao P, Zhu J, Cheng H, Cao J, Lu G. 2015. Biofilm Formation Caused by Clinical *Acinetobacter baumannii* Isolates Is Associated with Overexpression of the AdeFGH Efflux Pump. Antimicrob Agents Chemother 59:4817-4825.
4. Navidifar T, Amin M, Rashno M. 2019. Effects of sub-inhibitory concentrations of meropenem and tigecycline on the expression of genes regulating pili, efflux pumps and virulence factors involved in biofilm formation by *Acinetobacter baumannii*. Infect Drug Resist 12:1099-1111.
5. de Breij A, Gaddy J, van der Meer J, Koning R, Koster A, van den Broek P, Actis L, Nibbering P, Dijkshoorn L. 2009. CsuA/BABCDE-dependent pili are not involved in the adherence of *Acinetobacter baumannii* ATCC19606(T) to human airway epithelial cells and their inflammatory response. Res Microbiol 160:213-8.
